# Attention deficit hyperactivity disorder: A pilot study for symptom assessment and diagnosis in children in Chile

**DOI:** 10.3389/fpsyg.2022.946273

**Published:** 2022-08-04

**Authors:** Isabella Fioravante, José Antonio Lozano-Lozano, Diana Martella

**Affiliations:** ^1^Escuela de Psicología, Pontificia Universidad Católica de Chile, Santiago, Chile; ^2^Instituto de Ciencias Biomédicas, Instituto Iberoamericano Desarrollo Sostenible, Claustro Académico del Doctorado en Ciencias Sociales, Universidad Autónoma de Chile, Santiago, Chile; ^3^Departamento de Psicología, Universidad Loyola de Andalucía, Sevilla, Spain

**Keywords:** attention deficit hyperactivity disorder (ADHD), children diagnosis, symptomatologic assessment, ADHD in Chile, DSM-V, TDAH in Chile

## Abstract

**Background:**

Attention Deficit Hyperactivity Disorder (ADHD) is one of the most prevalent psychiatric disorders among school-age children and is characterized by varying degrees of inattention, hyperactivity, and impulsivity. Diagnosis, which currently relies on the DSM-V criteria, is complex. This research proposes an integrated procedure for ADHD diagnosis in children, improving the diagnostic process and scientific research on etiopathology.

**Materials and methods:**

We conducted a clinical report on ADHD diagnosis in children (*n* = 92) between the ages of 8 and 13, based on the results of the application of different scales to parents of school-age children in Chile. The children were divided into two groups, those with an ADHD diagnosis (*n* = 44) and those without (*n* = 48) (24% females).

**Results:**

The results revealed statistically significant differences between groups for scales EDAH y SDQ-Cas, Conners Comprehensive Behavior Scale, Conners Parent Scale and the criteria according to the DSM-V and its dimensions, with the exception of inattention.

**Conclusion:**

The findings indicate the importance of appropriate criteria and procedures to establish a diagnosis and implement effective interventions in ADHD.

## Introduction

Attention Deficit Hyperactivity Disorder (ADHD) is a neurodevelopmental disorder characterized by symptoms of inattention, impulsivity, and hyperactivity (with subtypes hyperactive-impulsive, inattentive, or combined), which interfere with development and impact the individual’s functional, personal, and social spheres (American Psychiatric Association, 2013), which could not be attributed to another neurological, sensory, language, or motor disorder. This is the main health problem that affects children, according to the most recent epidemiological data (Polanczyk et al., 2007, 2014; Thomas et al., 2015), and is the most frequent diagnosis in scholar children and adolescents, affecting their adaptation to the school environment. Children and young people with ADHD have a higher risk of school failures or delays, family conflicts, risk behaviors, substance abuse, among others (Leahy, 2017). Due to its clinical heterogeneity and the absence of a biological marker, the diagnosis of ADHD is currently complex (Drechsler et al., 2020; Martella et al., 2020; Sutcubasi et al., 2020).

According to the last review by Polanczyk et al. (2014), it is estimated that the worldwide prevalence rate of ADHD is between 6 and 7% among the population under age 18, slightly higher than the 5.3% estimated in another study from 2007 (Polanczyk et al., 2007). Although ADHD is configured as the most frequent neurodevelopmental disorder, few studies offer prevalence estimates regarding mental health pathologies such as ADHD in Chile. Thus, the benchmark study for Chile continues to be De la Barra et al. (2013) and was noted in the country’s most recent National Plan for Mental Health (2017–2025) as the source of prevalence data, but it focuses only on the epidemiological aspect of the disorder, especially yielding information to children and adolescent mental health programmes in Chile, while a variety of strategies to detect, diagnose and treat pathologies in health and education areas have emerged (Chile Ministerio de Salud, 2008; Chile Ministerio de Educación, 2015; Reyes et al., 2019).

Rendering to that study, the prevalence of ADHD in Chile is 10.3% among children ages 4–18, with the highest prevalence in ages four to eleven (15.5% nationally and 18.7% in Santiago), representing one of the highest prevalence in the world (Uribe et al., 2019). The most prevalent subtype is the hyperactive-impulsive, showing no gender differences, and the most prevalent comorbidities are anxiety disorders and oppositional disorder. Some correlations are relevant to point: the perception of a good and functional family have a negative association with ADHD diagnosis, while maltreatment has a positive correlation (De la Barra et al., 2013). Importantly, the reported prevalence is significantly higher than the overall prevalence in Chile, currently estimated at 7% (Thomas et al., 2015), and is also important to note that children and adolescents diagnosed with ADHD have high rates of consultation of mental health services (50.9%), compared to those with other disruptive disorders (27.6%) and non-disruptive disorders (36.8%) (De la Barra et al., 2013).

An ADHD diagnosis mainly relies on the criteria established in the Diagnostic and Statistical Manual of the American Academy of Psychiatry (DSM) (American Psychiatric Association, 2013). Both the DSM and other diagnostic classification manuals primarily group symptoms based on criteria to help the professional group different disorders. Therefore, these manuals constitute non-dimensional, consensus descriptive classifications and are currently multiaxial, as is the case of DSM-V. In other words, they are organized around different diagnostic axes that allow additional information relevant to the principal diagnosis to be included. However, all criteria continue to be clinical and only descriptive, despite ongoing efforts to standardize them and increase their objectivity. In keeping with the fifth version of the DSM, symptoms are given greater emphasis than the dysfunctionality; that is, the importance of symptoms corresponding to the dysfunction is diminished (Rojas et al., 2018).

This shows that the thresholds for classifying a child’s behavior as disproportionate will always be arbitrary to some degree; here factors such as the cultural norms of each context, an adequate knowledge of typical childhood development, and the expectations of parents and teachers all come into play. These differences led to discrepancies, which some authors have argued that the prevalence varies according to age and the number of symptoms observed by informants (McKeown et al., 2015). Moreover, importantly, this diagnostic procedure has been criticized for not allowing sufficient reliability or validity (Faraone et al., 2014). In this framework, there are currently studies that suggest differences in cognitive functioning depending on the ADHD subtype, which would mean that the associated clinical deterioration is heterogeneous (Rivera, 2016). For example, regarding attention processes, in the inattentive subtype of ADHD, the alteration of selective attention would be seen more frequently; while in the combined subtype sustained attention would be more affected. Due to the above, the gigantic methodological differences in the experimental studies make the revision of this matter an arduous task.

There is no standardized approach to integrating the multiple sources of information into an ADHD diagnosis. Nor does the recent fifth edition of the DSM offer this possibility, due to a lack of empirical data that would allow for the integration of all mechanisms that figure into a diagnosis (Martel et al., 2015). In fact, the increasing diagnosis of ADHD in children around the world has started many debates about the validity of the diagnosis process, including in the social sciences field, with debates especially concerning the diagnosis and treatment of ADHD from a children’s behavior “medicalization” perspective (Rafalovich, 2008; Hinshaw and Scheffler, 2014; Reyes et al., 2019).

Various studies based on neuroimaging and electrophysiological measurements have supported the hypothesis of ADHD’s neurobiological origin, although its exact etiology cannot yet be confirmed. It is also essential to consider that ADHD is a pathology with a high heritability rate, estimated at up to 80% (Faraone et al., 2014). Other studies using electroencephalography (EEG) technique have found controversial results, with no consensus on analysis of EEG frequency bands in ADHD subjects, and the likely reason for this lack of consistent results is the heterogeneity of ADHD subtypes and of tasks (Fabio et al., 2018). In Chile, some studies have presented evidence from the neurobiological aspects of ADHD (Aboitiz and Schröter, 2005; Aboitiz et al., 2012), and the more promising founds are related to deficit in the functioning of neurotransmitters, cerebral dysfunction in frontal structures and deficit in executive functions. Especially considering the clinical heterogeneity of ADHD children, they will probably exhibit a heterogeneous neuropsychological profile too (Fabio et al., 2018).

Many authors (e.g., Abad-Mas et al., 2017; Santana-Vidal et al., 2020) have proposed, even previous the DSM update from fourth to fifth edition (Barkley, 2009) that there are problems with the clinical application of the ADHD criteria. They refer especially to the extent of symptoms list and their operational definitions, calling for the need to review them, and most important, to integrate other measures specifically regarding the executive functions. Some authors have been interested in the hypothesis that children with ADHD have an underlying executive dysfunction, maybe due to an impairment of the automatic processing of basic skills (Martino et al., 2017), proposing that in addition to attention difficulties, there are other impairments that affects children with ADHD, such as memory, inhibition, and planning difficulties (Fabio, 2017).

Additionally, due to the heterogeneity of the disorder’s clinical presentation and the absence of a biomarker, professionals often resort to diagnosis by exclusion after assessing for other comorbid pathologies that present similar behavioral manifestations, as reported in the study by De la Barra et al. (2013), mainly anxiety disorder and oppositional-defiant disorder. There is rarely a “pure” presentation of ADHD, yielding a high rate of comorbidity with other conditions that can hinder an initial diagnosis of ADHD (Fenollar-Cortés and Fuentes, 2016). Between 40 and 80% of those with ADHD present some type of comorbid association (Aìlvarez et al., 2013). Therefore, it is typical for presentation of ADHD to occur in conjunction with another disorder (Orjales, 2012; Roessner et al., 2016; Liu et al., 2021).

Following that the state of the art about ADHD limit it diagnosis to the solely clinical analysis, in Chile the disorder is diagnosed by qualified professionals (which could be a neurologist, psychiatrist, pediatrician, general physician, psychologist, teachers’ differential behavior, or educational psychologist) in accordance with guidelines by the Ministry of Education and Ministry of Health (Decree number 170/2010). In short, it means that the diagnosis is made through the check of the criteria that encompasses the disorder.

The Chilean decree guidelines indicate that the diagnosis process follows a three steps protocol, which includes (1) the classification according to the most recent edition of diagnostic classification manuals, a (2) detection and assessing process based on criteria such Conners Test, and a (3) comprehensive diagnostic process including a diagnosis by exclusion review. To this point, it is important to note that the behavioral observation is made based on the Conners Test, which is a questionnaire that is widely disseminated on the internet and does not fulfill the international diagnostic recommendations (Santana-Vidal et al., 2020).

More specifically, the increase of ADHD diagnosis in Chile has become on 2000s, and led their incorporation into children’s health plan named “*Habilidades para la vida*” (Chile Ministerio de Salud, 2008), which aims are prevent this type of disorder through a joint work between school and health services, and has been accompanied by the creation of devices such as the School Integration Program (PIE) in 2015 (Chile Ministerio de Educación, 2015). This leads to another relevant issue: the fact that the diagnosis rate of “special educational need” disorders (defined by “PIE”) are the basis to a state subside to the schools, and since ADHD is one of them, they diagnosis can be used as a strategy to obtain additional economic resources, which has aroused different suspicions (Reyes et al., 2019). Surprisingly or not, the sophistication of these strategies has coincided with the sustained increase of ADHD prevalence rates on child and youth population (Uribe et al., 2019). In this work, ADHD is understood as a contingent pathology and a public and clinical health problem (Pelham et al., 2020), due to its transversal impact on the different areas of childhood development and the importance of clearly establishing its structure, etiology, and expression. This work seeks to point out a problem that has receive few attentions in Chile, and that constitutes a major problem around the world: the lack for an integrative and objective methodology for ADHD diagnosis, which could derive both in over and underdiagnosis. The current empirical scientific literature on this field, especially in Chile, is scarce and reveals the relevance of making this problem visible.

As a result, the aim herein was to propose a battery of instruments for an independent procedure of symptom analysis and diagnosis of ADHD: the Conners scales, EDAH, SDQ-Cas and the criteria established in the DSM-V. Furthermore, this work seeks to foster a discussion, especially in Chile, about the need for integrated diagnostic procedures in children, supported by the belief an objective, integrated diagnostic system is the best way to approach complex disorders such as ADHD. Such a system would contribute to standardized, enhanced diagnoses and also to scientific research into the etiological mechanisms of such disorders. Specifically in Chile, this work will contribute to present empirical evidence to support the claim to improve the current ADHD assess standard, in line with the current worldwide research.

## Materials and methods

This research relies on a pre-experimental design involving two non-randomized groups (ADHD and Normotypical) and a single measure (Chacón-Moscoso et al., 2008; Chacón-Moscoso et al., 2016).

### Participants

Ninety-two boys (*n* = seventy) and girls (*n* = twenty-two), between the ages of eight and sixteen (M = 11.07; SD = 1.561) participated in the study. Children were recruited in Chilean public schools catering to populations with a similar socioeconomic status, belonging to three educational establishments in the city of Talca and one educational establishment in the city of Santiago (Chile). These schools were characterized to implement the Integration School Program (PIE), where children with ADHD have a previous diagnosis from a psychiatrist. The schools were selected from a database of schools participating in other research projects. The inclusion criteria for this study were: (a) a diagnosis of ADHD and (b) no history of cognitive impairment, brain trauma, neurological disease, physical disability, comorbid mental disorders (except oppositional defiant disorder), or learning disorders. For the neurotypical group, participants were matched by gender, age, and IQ scores (in the case of the ADHD group). Any children presenting symptoms that could indicate ADHD were excluded. All children had an IQ above the 75th percentile, according to the results of Raven’s Colored and Progressive Matrices (Raven, 1976). The mean age and IQ scores of the two groups were not significantly different. For each child, a Hand Preference Index was assessed by means of a standard Lateral Preference Questionnaire.

According to the allocation criteria, 44 children were assigned to the ADHD group (Group 1) and 48 children to the neurotypical group (Group 2). No significant differences were found in most of the sociodemographic variables, except in the use of medication: χ^2^ (1, *N* = 92) = 17.502, *p* < 0.05. Table 1 shows the sociodemographic characteristics of the sample.

**TABLE 1:** Students and parents’ characterization (*N* = 92).

Research variables	ADHD-G *N* = 44	N-G *N* = 48	Test	*P*
Children’s gender			χ^2^	0.07
Female	5 (11.4)	17 (35.4)		
Male	39 (88.6)	31 (64.6)		
Age			U	0.063
Mean (SD)	10.77(1.428)	11.33(1.642)		
Median (IQR)	10(1)	12(3)		
Min-max	9-15	8-16		
Childbirth			χ^2^	0.146
Term	41 (93.2)	40 (83.3)		
Premature	3 (6.8)	8(16.7)		
Birth type			χ^2^	0.883
Normal	19 (43.2)	20 (41.7)		
Cesarean section	25 (56.8)	28(58.3)		
Problem during birth			F	0.511
Yes	6 (13.6)	4(8.3)		
No	38 (86.4)	44 (91.7)		
Sleep disorder			χ^2^	0.211
Yes	5 (11.4)	3 (6.3)		
No	37 (84.1)	45 (93.8)		
Medication use				
Yes	27 (61.4)	9 (18.8)	χ^2^	0.001
No	17 (38.6)	39 (81.3)		
Mother education			U	0.625
Elementary/Middle	14 (31.8)	15 (31.3)		
High school	24(54.5)	23 (74.9)		
College	6 (13.6)	10 (20.8)		
Father education			U	0.122
Elementary/Middle	25 (56.8)	20 (41.7)		
High school	15 (34.1)	20 (41.7)		
College	4 (9.1)	8 (16.7)		

ADHD-G, ADHD group; N-G, neurotypical group; χ^2^, Chi-square; U, Mann-Whitney U; *F*, Fisher test.

### Measures

Standard instruments validated for a Spanish-speaking population were used, except for the anamnesis form and the ADHD diagnostic checklist, which the authors developed for the specific purposes of this study.

1. Anamnesis Record: an *ad hoc* instrument composed of nine elements, which records information provided by the parent/guardian regarding the birth history, development, and health of the child, and sociodemographic characteristics of the family unit.
2. Conners Scales (Conners, 1970, 1989, 1997; Farré and Narbona, 1989): the adaptations for the Spanish population of the Comprehensive Behavior Ratings Scale (α = 0.94; extended form with 48 items) and the Parents Rating Scale (α = 0.90; abbreviated form with ten items) adapted by Farré and Narbona (1989) were used. These scales collect reported information to identify behavioral changes and symptoms of ADHD.
3. Scale for the Evaluation of Attention Deficit Hyperactivity Disorder— EDAH-, its Spanish acronym (Farreí and Narbona, 2000; Belmar et al., 2015): validated for the Chilean population (α = 0.95) by Belmar et al. (2015), it consists of 20 items and aims to assess the main features of ADHD and any coexisting behavioral disorders.
4. The SDQ-Cas questionnaire (Goodman, 1997; Brown et al., 2014): a study of psychometric properties among the Chilean population (α = 0.79; Brown et al., 2014), it consists of 25 items that gauge behaviors, emotions, and interpersonal interactions associated with psychological problems in children and adolescents. In addition, the impact supplement (on the reverse side of the questionnaire) enables professionals to ask parents if the child shows any type of problem covered in the scales, with another series of questions regarding chronicity, distress, social impairment, and the burden to others that behavioral problems can generate.
5. Attention deficit hyperactivity disorder diagnostic checklist: the ADHD diagnostic criteria defined in the DSM-V (American Psychiatric Association, 2013) and used by the Chilean Ministry of Health were applied. The criteria were converted into a table and applied as a checklist consisting of three sections (inattention, hyperactivity, other criteria) and the total (sum of presence/absence of all criteria).

### Procedures

This study is part of a FONDECYT–project (1181472) and obtained ethical approval by the National Agency for Research and Development (ANID) of Chile. The Research Ethics Committee of the Autonomous University of Chile also approved the study (approval number 012–2019).

First, an invitation was extended to each school to participate in the research. Once the school principal had provided informed consent, the project was overseen by the research team in conjunction with the directors of technical-pedagogical units, school integration programs, or other pertinent professionals. Then, the parents of the children who were potential participants were contacted to respond to the battery of instruments used to characterize the children. After this stage of evaluation, the data were screened according to inclusion and exclusion criteria, and the pertinent statistical analyses were carried out.

### Data analysis

The assumptions of normality and homogeneity of variance were verified, and all variables followed a non-normal distribution except for the EDAH and the SDQ-CAS scales.

Version 26.0 of the statistical package SPSS was used for the descriptive calculations and contrasts of means. And for the estimation of statistical power and effect size, the GPower version 3.1 package was used. To compare the means between the groups, a minimum significance level of 0.05 was considered. The confidence intervals in the estimates of the parameters were 95%. Normality assumptions were verified using the Shapiro-Wilk test (normal distribution assumed *p* > 0.05); linearity was checked (met when *p* < 0.05); and error independence was verified with the Durbin-Watson test (values between 1.5 < d < 2.5 were considered adequate). Since not all assumptions were satisfactory, Spearman’s bivariate correlations (ρ) were calculated. In those cases where the chi square cannot be applied, Fisher’s exact test was used. The Mann-Whitney U statistic was used in all cases except those that fulfilled the assumption of normality and homoscedasticity, in which case Student’s *t* statistic was used. Cohen’s d was used to calculate the effect size based on the differences.

## Results

In order to assess any significant differences between the ADHD group and the neurotypical group in each of the variables, a means comparison analysis was carried out. As seen on Tables 2, 3, all study variables showed significant differences, except for the inattention dimension of DSM.

**TABLE 2:** Differences between groups for scales means (excepting EDAH and SDQ-Cas) (*N* = 92).

Measure	Group	N	Rank	z	U	*p*	1-β	d
DSM inattention	ADHD-G	44	49.01	−0.886	945.500	0.376	0.058	0.0583
	N-G	48	44.20					
DSM hyperactivity	ADHD-G	44	57.19	−3.710	585.500	0.001	0.833	0.833
	N-G	48	36.70					
DSM other criteria	ADHD-G	44	55.86	−3.496	644.000	0.001	0.913	0.718
	N-G	48	37.92					
DSM total	ADHD-G	44	55.44	−3.083	662.500	0.002	0.871	0.667
	N-G	48	38.30					
Conners scale parents/custodians	ADHD-G	44	55.07	−2.952	679.000	0.003	0.873	0.670
	N-G	48	38.65					
Conners scale home behavior	ADHD-G	44	54.61	−2.792	699.000	0.005	0.824	0.625
	N-G	48	39.06					

ADHD-G, ADHD group; N-G, neurotypical group; U, Mann-Whitney U; 1-β, statistical power; d: effect size.

**TABLE 3:** Differences between groups for EDAH and SDQ-Cas—scales means (*N* = 92).

	ADHD-G *N* = 44		N-G *N* = 48						
	M	DE	M	DE	gl	t	*p*	1-β	d
EDAH	28.75	12.908	20.12	11.452	90	3.397	0.001	0.957	0.707
SDQ-CAS	25.82	4.962	21.58	5.119	90	4,022	0.001	0.983	0.793

ADHD-G: ADHD group; N-G, neurotypical group; *t: t* student; 1-β, statistical power; d, effect size.

Likewise, a bivariate correlation analysis was performed using Spearman’s test to examine differences between the instruments (see Table 4). The results show a statistically significant correlation between the instruments, except between the inattention dimension of the DSM-V and the dimensions of hyperactivity, other indicators, and the SDQ-Cas test.

**TABLE 4:** Correlation coefficients between instruments (Spearman’s Rho).

	1	2	3	4	5	6	7	8
1. DSM-inattention	−							
2. DSM-hyperactivity	0.187	−						
3. DSM-other criteria	0.188	0.293**	−					
4. DSM-total	0.697**	0.638**	0.412**	−				
5. EDAH	0.417**	0.500**	0.215*	0.615**	−			
6. Conners-P/C	0.383**	0.436**	0.294**	0.516**	0.823**	−		
7. Conners-H/B	0.371**	0.431**	0.240*	0.537**	0.760**	0.839**	−	
8. SDQ-CAS	0.189	0.381**	0.246*	0.411**	0.508**	0.487**	0.645**	–

*N* = 92. Spearman’s correlations are shown. **p < 0.01; *p < 0.05.

## Discussion

Attention deficit hyperactivity disorder has been the subject of a plethora of studies and reviews that have led to changes in its diagnosis and treatment over the years. Although its symptoms have been known for centuries, it has only been recognized as a pathology in children since the 1980s and adults since 2013 (APA). Since then, ADHD has become one of the most extensively studied—yet also one of the most controversial—disorders (Wolraich, 1999). Therefore, the role of those involved in the suspicion, diagnosis, and intervention in cases of ADHD becomes exceedingly relevant since they are in positions of power that allow core practices to be instilled and/or reinforced. Increasingly rigorous research on determining whether a child may have ADHD thus becomes crucial.

The results of the application of the clinical tests in this study demonstrate the discrimination capacity of the instruments used for the evaluation and diagnosis of ADHD. One interesting finding was the results of the SDQ-Cas, consistent with prior scientific literature indicating its ability to discriminate and suggesting it may be helpful during a diagnosis as a supplementary indicator of ADHD. In turn, the analyses of the detailed results of the DSM-V criteria showed that the hyperactivity dimensions and other indicators were statistically significant. This was not the case for inattention, which suggests that this indicator is not determinant in a diagnosis of the disorder and should be considered jointly with other markers that confirm a hypothesis of ADHD.

Finally, the correlational analyses revealed significant direct effects for the correlations between most of the instruments. This indicates the usefulness of combining the instruments to enhance the process of diagnosing ADHD, which, in turn, ratifies the importance of defining a diagnostic protocol.

By contributing to the discussion on the evaluation and diagnosis of ADHD, this article set out to demonstrate the importance of establishing appropriate mechanisms to ensure that children receive a correct assessment and diagnosis regardless of their sociodemographic characteristics. Based on the findings herein and scientific advances in this field, there is a need for a protocol that can render professional practice more effective and standardize it for children with ADHD.

Children’s families and schools are generally the first to distinguish the symptoms of ADHD, which is why most of the instruments used for a diagnosis involve an initial assessment of the child’s behavior by their teachers and parents (Garcia-Rosales et al., 2020). Also, it is likely that the high expectations of school performance, which parents and teachers place on children, increase the need to find clinical explanations for school failure (Santana-Vidal et al., 2020). However, according to different studies, parent-teacher agreement on ADHD symptoms has typically been low to moderate (Narad et al., 2015). In this sense, it is also important to acknowledge that the current assessment method has been criticized for a lack of diagnostic precision and even differences between the most widely used manuals (ICD-10; World Health Organization [WHO], 1992; and DSM-V).

In this regard, the relevance of the present study becomes apparent. The results reveal the need for an assessment alternative that allows for greater procedural objectivity and a diagnosis based on more integrated appraisals of ADHD symptoms. The new assessment process could incorporate, for example, experimental tests that can reduce the time of an ADHD diagnostic procedure and increase its accuracy (Hall et al., 2016). Likewise, neuropsychological tasks can contribute and complement the behavioral measures (Santana-Vidal et al., 2020).

Diagnosing ADHD is a delicate task that is the subject of much debate around its etiology and, consequently, its symptoms. Therefore, it is a multifactorial disorder and needs to be addressed as such. Regarding future research, this work suggests that more investigation is needed into the changing diagnostic criteria of the main classification manuals, the evolution of how the disorder is conceptualized, and international differences in its assessment. This is crucial because one of the possible causes of overdiagnosis and underdiagnosis is the existence of ineffective instruments (Santana-Vidal et al., 2020). Moreover, the confounding criteria for the diagnosis of ADHD represent an issue that makes detection difficult, especially if it is carried out by professionals with little experience in the area (Ferrer-Urbina et al., 2017).

Children with ADHD represent a heterogeneous population and vary greatly in the degrees and severity of symptoms (Leahy, 2017). Follow-up studies with samples of ADHD children have showed that they have a higher-grade retention rate, more participation on special educational needs programs, school suspensions, more school expulsion, and lower academic performance, compared with control groups (Pi et al., 2018). This evidence makes such investigation as proposed in this present work all the more pressing.

Considering all that has been presented herein, the implication of this study relies on both clinical and practical areas. Regarding the clinical implications, on one hand, the construction of an integrative theoretical model for ADHD, incorporating hypotheses that support the biological, genetic, environmental, cognitive, and emotional factors that compose it, will impact directly on the comprehension and the handling of the disorder, for all those health professionals who have the power of the assessment and diagnosis process. On the other hand, regarding the practical implications, a change on the conception of ADHD diagnosis and the availability of a protocol to guide the practice will have a strong impact on the daily basis of several educational professionals and, therefore, on many families who put their trust in them.

## Limitations

The present study presents some limitations in its execution. One of the main limitations of the study is the sample size which makes it difficult to generalize the findings obtained. Our procedure consisted of collecting data for 1 year. Initially we proposed to incorporate more schools, but due to the social unrest in Chile, the end of 2018, followed by the COVID-19 outbreak, it became impossible for us. Although the results obtained are representative of the schools being evaluated, in the immediate future we hope to incorporate more schools and consider including other age ranges, given that attentional capacity varies according to developmental stages (Milani et al., 2022).

Another limitation presented by the study is that the anamnesis form and the ADHD diagnostic checklist have not been validated for the Spanish-speaking population. For future developments, when the sample size allows, we will conduct validity and reliability studies. Specifically: validity evidence based on test content. Expert specialists will examine whether the proposed items are relevant, useful, and feasible (Chacón Moscoso et al., 2019). Evidence based on construct validity (Holgado-Tello et al., 2018) considering all the stages of validation (Muñiz and Fonseca-Pedrero, 2019). The developments of this line of work would allow the standardization of tests in Chilean educational contexts that facilitate the application of ADHD symptom evaluation protocols.

## Conclusion

Because ADHD is the most common behavioral disorder of childhood, an appropriate and sensitive evaluation of symptoms is essential (Rostain et al., 2015). Even so, guidelines used for diagnosis of ADHD are not still rigorously applied, leading to an underdiagnosis or overdiagnosis of ADHD (Manos et al., 2017). The main finding of this study indicated that the application of the clinical tests to parents of children whit ADHD diagnosis appoints to the discrimination capacity of the instruments used for the evaluation of symptoms of ADHD. This first evaluation could be very relevant as a useful guide for clinicians in the diagnosis of ADHD.

Finally, the findings of this study will allow for the implementation of essential considerations in the assessment and diagnosis of children with ADHD and contribute to advancing the discussion in the scientific community.

## Data availability statement

The raw data supporting the conclusions of this article will be made available by the authors, without undue reservation.

## Ethics statement

The studies involving human participants were reviewed and approved by Comité Ético Científico de la Universidad Autónoma de Chile. Written informed consent to participate in this study was provided by the participants’ legal guardian/next of kin.

## Author contributions

The initial idea was generated by DM and IF and developed by all authors. IF collected all the information. JL-L analyzed the data. All authors wrote the manuscript, made a substantial contribution to the design of the document and improving both its writing and structure, consented to this final version for publication, and agreed to be responsible for all aspects of the work, such as the accuracy of the data and the completeness of the investigation.
